# Communicating unexpected news to pregnant people living with mental health conditions in fetal medicine (the UNDERSTAND study): Healthcare professionals’ perspectives

**DOI:** 10.1371/journal.pone.0347547

**Published:** 2026-05-15

**Authors:** Ioannis Karapanos, Iona Hindes, Jemima Dooley, Nikolina Jovanović, Maddalena Miele, Jane Fisher, Stamatina Iliodromiti, Elena Greco

**Affiliations:** 1 Women’s Health Research Unit, Centre for public Health and Policy, Wolfson Institute of population health, Queen Mary University London, England; 2 Sir Henry Wellcome Centre for Mood disorders Research, Exeter University, England; 3 Centre for Psychiatry, Wolfson Institute of population health, Queen Mary University London, England; 4 Perinatal Mental Health services, East London NHS Foundation trust, England; 5 Imperial college London, England; 6 Antenatal Results and Choices, Registered charity No 1148663, London, United Kingdom; National Institute of Mental Health and Neurosciences: National Institute of Mental Health and Neuro Sciences, INDIA

## Abstract

**Objectives:**

This study explores the perspectives and experiences of healthcare professionals (HCPs) in a multidisciplinary fetal medicine (FM) team when delivering unexpected news in fetal medicine, with a particular focus on pregnant people living with mental health conditions (MHCs). It aims to identify communication strategies and highlight facilitators and barriers to effective consultations in these challenging situations.

**Methods:**

Qualitative study involving 20 semi-structured interviews with HCPs from multi-disciplinary FM teams in a large NHS Trust in East London. Participants included FM specialists, neonatologists, midwives, and specialist nurses. Data were analyzed using reflexive thematic analysis to identify key themes representing participants’ perspectives.

**Results:**

Five key themes emerged: (1) enhancing patient understanding, (2) supporting patients (3) individualizing care, (4) supporting HCPs and (5) the impact of health inequalities in FM. Facilitators of effective communication included optimizing the setting, avoiding ambiguity, fostering understanding, and involving significant others in discussions. Barriers included language limitations, where reliance on interpreters hindered effective and compassionate communication, and poor pregnancy-related health literacy, particularly in socio-economically deprived populations, which impeded informed decision-making. Clinician-related barriers included reluctance to address MHC due to lack of training, stigma around mental health, and time constraints. The emotional toll of frequently delivering distressing news was identified as a significant challenge for HCPs, yet formal support mechanisms were lacking.

**Conclusion:**

HCPs in FM emphasized that delivering unexpected news requires a patient-centred approach that considers individual needs, is culturally sensitive, and addresses language and health literacy barriers. The mental health and well-being of both those receiving and delivering the news remains largely overlooked. Currently, no specific communication training exists for HCPs in FM and limited or no support is available for individuals with mild, pre-existing, or suspected MHC, despite their prevalence and the recognition that difficult news during pregnancy may exacerbate these conditions. Future research should evaluate communication frameworks co-produced by HCPs and individuals with lived experience to improve FM consultations for patients with and without MHC. Enhanced mental health support is urgently needed for both patients and HCPs in FM.

## Introduction

Unexpected news is defined as any information that negatively alters a person’s expectations regarding their present and future [1]. In the pregnancy context, this includes unforeseen complications that may affect the mother, fetus, or the anticipated course of pregnancy [2–4]. Unexpected news may cause significant distress for both the recipients and those delivering it. For prospective parents, it often elicits a wide range of emotional responses [5] and may impact their mental well-being [6]. Healthcare professionals (HCPs) tasked with delivering such news also experience considerable emotional strain, with high rates of poor mental health reported among those frequently engaged in difficult conversations [7]. Notably, research suggests that inadequate communication skills may lead HCPs to withhold information to minimize patients’ distress, potentially limiting parental understanding and impairing informed decision making [8]. Conversely, good communication skills have been shown to positively influence recipients’ emotional responses, and enhance their ability to make informed choices [9]. Historically, literature had focused more on the optimal way to deliver such news. However, in more recent years research studies have also recognized the importance of navigating patients through the difficult conversations that take place after the initial delivery of such news [10].

In fetal medicine (FM), delivering unexpected news is a routine but challenging aspect of practice [11], often involving complex health information with uncertain prognosis, that requires difficult decisions, or devastating news such as intrauterine fetal demise. However, comprehensive guidance and training in this area remains inadequate [12,13]. Existing communication frameworks are often generic, developed primarily by and for doctors, with limited focus on patient outcomes [14]. Previous research on difficult conversations in antenatal ultrasound, has examined the views of specific professional groups, such as sonographers [15–17] or obstetricians [18]. Studies exploring the experiences of diverse HCPs and addressing also the emotional burden of delivering news are scarce [19,20].

Poor perinatal mental health is an increasing concern, with up to one in five women in the UK getting diagnosed with a mental health condition(MHC) during pregnancy and the puerperium. [21]. The World Health Organization’s International Classification of Diseases (ICD-11) defines mental, behavioral, and neurodevelopmental disorders as conditions characterized by clinically significant disturbances in cognition, emotional regulation, or behavior [22]. These conditions can vary in severity, ranging from mild to moderate or severe, and may impair an individual’s ability to process complex information, regulate emotions, and make informed decisions [22,23]. Consequently, communicating unexpected news to pregnant people with pre-existing MHC may require additional sensitivity and tailored communication strategies to ensure understanding and support decision-making. Despite this, the specific needs of this population in clinical communication remain under-explored.

Most research on communicating with individuals with MHCs has focused either on delivering diagnosis, such as schizophrenia, or on communication within psychotherapy settings [24–26]. Although, some practical strategies to improve clinician-patient communication with people with MHCs do exist [27], these approaches may not be directly applicable to pregnant women.

The “Unexpected news in pregnancy: a co-design study between service users and healthcare professionals to improve communication and decision-making in women with MHC (UNDERSTAND)” project seeks to improve patient experience in FM through the co-development of a communication framework (protocol in supporting information – S1 File). It uses a triangulation technique by drawing on filmed FM consultations, interviews with FM HCPs, and insights from people with lived experience of receiving unexpected news in pregnancy. In this paper, we present findings from interviews exploring healthcare professionals’ perspectives on the delivery of unexpected news in FM, including how communication strategies may need to be adapted when patients have known or suspected MHC.

## Methods

### Study design

This study employed an inductive qualitative approach using semi-structured interviews. This method is particularly suited for addressing questions such as “how” and “why”, and capturing emotions and reactions, concepts that quantitative methods may not fully explore [28]. Additionally, it is highly effective for exploring sensitive topics, as it allows flexibility and responsiveness to participants’ narratives.

Our methodological framework is grounded in relativism-constructionism, an epistemological theory asserting that knowledge is constructed through social interactions and shared experiences. Within this ontological perspective, the researcher’s life experiences inform data interpretation and meanings are co-created with participants [29].

### Study population and recruitment

We recruited HCPs from a large Trust in central London which encompasses three FM units: one tertiary referral center and two local units. These units serve a highly diverse and socially deprived population. From 15^th^ of April 2023 until 15^th^ of December 2023, the study team promoted the research among the HCPs of the FM multidisciplinary team (MDT) through meetings and departmental email communication (inclusion criteria in supporting information). All staff were invited to participate voluntarily; no individuals were approached directly. This approach was intended to reduce perceived pressure to participate, and to support open, candid engagement. Recruitment was purposeful to ensure diverse representation across demographics, roles, grades, and sites, capturing a broad range of experiences and perspectives. The recruitment target of 20–30 participants was guided by the qualitative concept of “information power”, which considers factors such as the study aim, sample specificity, theoretical framework, quality of dialogue, and analysis strategy [30]. Guided by the principles of reflexive thematic analysis, final decisions about adequacy of the sample size were made iteratively during data collection, based on the emerging quality, complexity and richness of the collected data [31].

### Data collection & transcription

Interviews were conducted between September 2023 and December 2023 by one author (IH), a social scientist with experience in interviewing on sensitive reproductive health topics. By selecting an interviewer with a non-clinical background and no supervisory or peer relationship to participants, we aimed to reduce potential response bias and mitigate power imbalances, particularly for junior staff. In doing so, perceived professional judgment during discussions of clinical decision making was minimized. Interviews were conducted face-to-face or virtually, depending on interviewee preference, and were audio recorded. A topic guide, co-designed by the research team and approved by the study’s Steering Committee, loosely structured the interviews. The guide broadly explored experiences of communicating unexpected news during pregnancy, including perceived barriers, facilitators, and opportunities for improvement. In addition, HCPs were specifically asked how they approach communication with patients who have MHC.

Interviews were transcribed verbatim by one author (IK), and transcripts were anonymised from the point of transcription. Identifiable details, including names and locations, were removed before analysis. In recognition of the small professional context, care was taken to redact or generalise any potentially identifying descriptions, particularly those involving rare clinical scenarios. Broad role descriptors (e.g., ‘midwife’, ‘FM specialist’) were retained to preserve analytic context while maintaining participant confidentiality.

### Data analysis – Reflexivity

Data were analysed using reflexive thematic analysis, following Braun and Clarke’s six-phase approach. [32]. In line with the interpretive orientation of reflexive thematic analysis, coding was conducted by a single author (IK) using initially an inductive, semantic approach and shaped by their active engagement with the data. Multiple independent coders or member checking were not used, as this is not aligned with the epistemological assumptions of reflexive thematic analysis, which emphasizes the researcher’s active role in interpretation rather than seeking inter-rater reliability [33].

IK has a background in FM and in order to recognize potential biases related to personal professional experiences, a reflexive journal was maintained throughout the analytic process. Regular reflexive discussions with other team members [JD, experienced qualitative researcher, EG, clinician with expertise in FM and JF, member of the public with extensive background in antenatal counselling], provided opportunities to reflect critically on researcher positionality, challenge assumptions, and explore alternative interpretations. These collaborative discussions helped the analysis evolve iteratively, with themes gradually refined to represent patterns of shared meaning across the dataset. The analytic process was non-linear, involving repeated movement between transcripts, codes, and candidate themes to ensure that final themes were grounded in the data and closely reflected participants’ perspectives [33,34].

### Ethical considerations

Ethical approval was obtained from the Social Care Research Ethics Committee (IRAS 310337). A Steering committee, comprising FM specialists, perinatal psychiatrists, obstetricians and midwives with special interest in mental health, psychologists and members of the public with lived experience, monitored the study’s progress and adherence to protocol, particularly with regards to ethical oversight and safeguarding support. Measures to safeguard participants’ emotional wellbeing were implemented, as recommended by the Steering Committee. All participants provided informed written consent.

## Results

Twenty eligible HCPs consented to participate in the interviews. Age, ethnicity, gender, role and years of experience varied widely amongst participants. Eighteen interviews were conducted online (Microsoft Teams) and two face to face. Mean interview duration was 45 minutes (range: 30–70 minutes).

**Fig 1:**
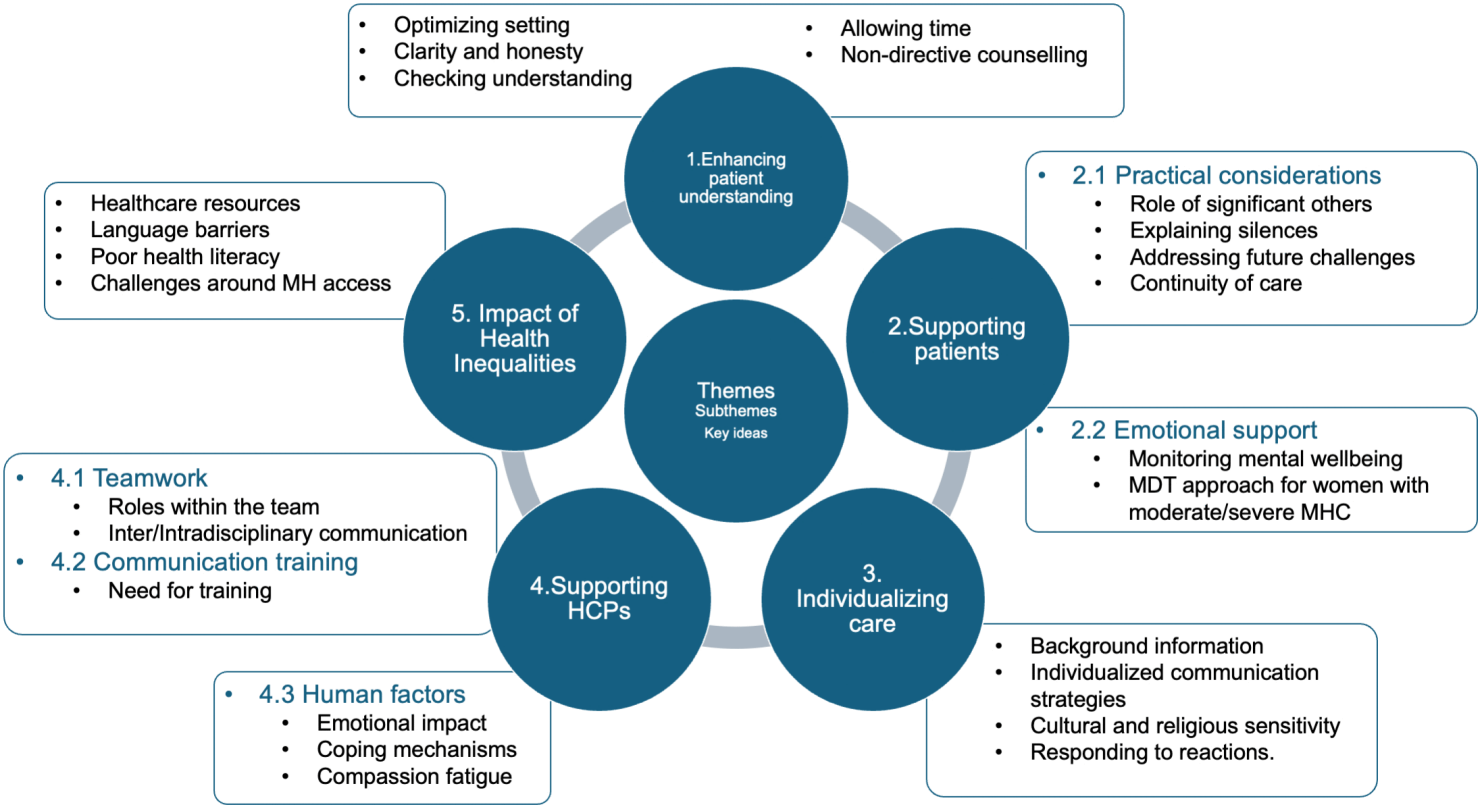
Overview of the themes and subthemes generated from the analysis, with associated key ideas.

Our analysis identified five main themes and corresponding sub-themes (Fig 1). Each theme is supported by key ideas and illustrative quotes. Quotes that reflect perspectives shared by a larger number of participants are presented in the Results section. All recommendations and additional supporting quotes are provided in Supporting Information - S2 File (Tables 1–5).

### 1. Enhancing patient understanding

This theme includes specific aspects of the process of news delivery considered crucial for optimising patient understanding of the information received (Table 1 - S2 File).

*Optimizing setting* Participants emphasized that both physical and non-physical aspects of the setting play a crucial role in how information is received. One participant described their approach: *“I would sit them down in front of me, so a woman is not lying down, and we have eye contact, and then I tell them that I’m very sorry, but I’m going to be direct” (#18, FM fellow).* Many preferred moving patients to a quiet room after the ultrasound examination, suggesting that a circular seating arrangement, rather than with an examination bed in between, could improve patients’ engagement. However, some acknowledged that initiating discussions during the ultrasound may be appropriate when specific diagnostic details need to be shown on the screen.

*Prioritizing clarity and honesty:* Participants favored a direct and honest communication style to help prospective parents prepare for future challenges and to enhance informed consent when multiple management options are available. Honesty was considered particularly important in cases of uncertain prognosis. One participant explained: *“...* [it is necessary] *being completely honest about what you can be certain about or not; there are situations where you have to say: “ we’re not sure what these findings mean”* (#9, FM consultant). Using plain language, avoiding medical jargon, and avoiding complex statistics were also highlighted as crucial for ensuring patient understanding of complex diagnoses and their implications.

*Checking understanding:* Interviewees acknowledged that while health information may seem routine for them, it is often complex, new and overwhelming for patients. They emphasized the importance of checking patients’ understanding before discussing management options. As one participant noted *“…it’s really important to take the time and check what their understanding is and only then you can go on to talk about the next steps…”* (#12, FM midwife).

*Allowing time for processing information:* Participants identified the need for patients to absorb information at their own pace as essential for understanding and decision-making. One interviewee suggested *“…You try to give information in very small chunks and then see how much they* [the patients] *can cope with…It’s not always about pouring in [delivering] all the information you have in one go…”* (#10, FM consultant). Many recalled instances where patients appeared overwhelmed, necessitating a pause in the conversation and arranging a follow-up discussion to allow time for processing and reflection.

*Non-directive counselling:* Clear, compassionate communication and non-directive counselling – where the patient rather than the HCP takes the lead, by asking questions and expressing concerns – were highlighted as key to support free, informed decisions. As one participant stated: *“I try to get across to parents that it’s their decision and we don’t have any judgment on them for what choice they make…” (#19, fetal cardiology consultant).*

### 2. Supporting patients

This theme includes key ideas around making patients feel supported throughout their pregnancy journey, and it is organized into two main sub-themes. The first addresses practical considerations for supporting patients during FM consultations (Table 2.1 – S2 File), while the second focuses on providing emotional support (Table 2.2 – S2 File).

#### 2.1. Practical considerations.

Role of significant others: Participants described significant others as crucial during difficult consultations to provide emotional support, reduce anxiety, help patients to process information, and advocating for patients who may withdraw. However, some challenges in involving them were noted: “…sometimes I will see that everyone is sat in the room and the patient might be there by herself and I’ll say, ‘do you have anyone with you’ and she’ll be like, ‘Oh yeah, my partner’s downstairs with the car’ and I’ll be like,’ OK, maybe we should get him to come’. And I don’t think that everyone always thinks of that because everyone’s so busy. And they’re like, ‘oh, God, I’m not waiting 15 minutes for her husband to get here’...” (#16, fetal cardiology specialist nurse). This highlights the need to prepare patients for the nature of discussions in FM consultations and encourage them to bring a support person.

*Explaining Silences:* Many interviewees noted that unexplained silence during ultrasound consultations can heighten patient anxiety. One participant explained: *“Because while they* [the patients] *are sitting and waiting with you saying nothing, that’s terrible and they’re getting more and more anxious.” (#15, FM consultant)*. To mitigate this, participants recommended pre-emptively addressing and explaining silences to prevent patients from feeling abandoned or ignored.

*Addressing future challenges:* Preparing patients for potential challenges ahead was seen as critical for supporting decision-making. Participants emphasized the importance of addressing, in simple terms, practical aspects of caring for a baby with health issues, including hospital stays, surgeries, complications, and possible outcomes, including worst- and best-case scenarios. One interviewee elaborated: *“My concern tends to focus more on ‘How are you going to cope with a baby in intensive care having open heart surgery in the first week of their life?’ or ‘What support can we put in place for when your baby is born?’ or ‘What can we do to plan ahead for how you’re going to take your baby home?’” (#16, fetal cardiology specialist nurse).* The importance of joint consultations with relevant HCPs and signposting to additional resources and peer-support groups was also highlighted.

*Continuity of care:* Many participants declared that continuity of care is essential for building trust and reducing patient distress. They also reported on patients expressing frustration about having to repeatedly explain their concerns to different HCPs. One interviewee noted: *“…a familiar face doesn’t make the problem go away, but it’s nice to see someone that you have seen before and who knows your history, so you don’t have to repeat things again.” (#14, FM midwife).*

#### 2.2. Emotional support.

*Monitoring Mental Well-Being:* Participants recognised that unexpected news can negatively impact mental wellbeing, regardless of a patient’s history of MHC. Acknowledging emotional distress and regularly monitoring patients’ emotional well-being were seen as crucial aspects of patient care. One participant shared their approach: *“…I usually call them a few days later and if it felt appropriate, I would say: ‘would you like me to refer you to our psychology team?’” (#16, fetal cardiology specialist nurse).* These steps were considered vital in identifying patients who may benefit from early referral to perinatal mental health services.

*Multidisciplinary Approach for Patients with Known MHC:* For patients with moderate to severe MHC, participants unanimously advocated for an MDT approach. One interviewee stated: *“I think it shouldn’t be just the FM team. I think it should be the FM team with the perinatal mental health team, or the consultant who’s involved and the midwives and then to have a proper MDT.” (#10, FM consultant).*

### 3. Individualizing care

Participants emphasized that a *one-size-fits-all* approach is inadequate for delivering unexpected news. This theme includes key ideas for tailoring care to reflect patients’ cultural backgrounds, preferences, and needs (Table 3 -S2 File).

*Gathering background information:* Our participants highlighted the importance of gathering information about the patient background as critical to ensure consultations can align to their individual needs. One participant explained: *“ I always try to understand who the patient is. Usually, I like to know what they do professionally or what they like to do, because that tells me what type of communication they will need; If they’re more visual, if they like numbers….” (# 17, FM consultant).*

*Individualized communication strategies:* Participants emphasized the importance of tailoring communication strategies to match the patient’s preferred approach to information gathering. Striking a balance between providing enough detail for informed decision-making without overwhelming patients was considered crucial. One interviewee suggested: *“Some people want a lot of information. Some people want very little information, and I think part of our role is to deliver enough information to whichever target people want and then give them the opportunity to ask questions and escalate information further thereafter” (#4, neonatal consultant).* Additionally, participants stressed the importance of considering each particular case holistically by discussing practical aspects and exploring available support systems as needed.

*Cultural and religious sensitivity:* Sensitivity to cultural and religious beliefs was recognised as essential for providing respectful and appropriate care, especially when it comes to sensitive topics like pregnancy termination. However, our participants recognised the importance of avoiding assumptions about patient preferences based on sociocultural backgrounds and ensuring that management options are presented clearly and impartially. One interviewee remarked*: “..Working in this community I’m trying to be a little bit gentle with this [discussing the option of medical termination of pregnancy]...sometimes I’d say ‘people are very different, and some couple would, some wouldn’t cope with a sick baby, so they would choose to go for termination… but for some couples it’s not an option”” (#17, senior FM fellow).*

*Clinicians responding to patients’ reactions:* Participants acknowledged the need to be prepared for a range of emotional reactions, including silence, distress, and agitation, when delivering unexpected news. Recognizing these responses as part of the grieving process and responding to them appropriately was considered essential. One interviewee shared*: “I’m prepared for the reactions, and I understand them as part of the process. So, some patients go silent, they sort of don’t say anything, some patients shout, and they become very distressed, agitated. Some patients want a second opinion because they are a bit in disbelief.” (#17, FM consultant)*. Several interviewees suggested that pausing consultations when patients become highly stressed could be beneficial, allowing the conversation to resume at a follow-up visit. In cases where patients request a second opinion due to disbelief or denial, participants agreed that such requests should be accommodated.

### 4. Supporting healthcare professionals

Fulfilling the needs and safeguarding the wellbeing of HCPs emerged as a recurrent theme in our interviews. This umbrella theme is organized into three subthemes: the importance of teamwork (Table 4.1 – S2 File), the need for HCP training (Table 4.2 – S2 File) and human factors in healthcare (Table 4.3 – S2 File)

#### 4.1. Teamwork.

*Roles within the team:* Doctors acknowledged that they often do not inquire about patients’ mental well-being during FM appointments, as their focus is primarily on clinical aspects. Additionally, the stigma surrounding MHC may prevent patients from openly discussing these concerns. Despite this, participants recognized the importance of emotional and psychological support, noting that midwives and specialist nurses are better placed to provide this care. As one participant remarked: *“Midwives often have much more of a supportive relationship* [with the patient]*. I’m the person who gives the horrible news and gives the facts and figures. But we do it together and we’ve got a nice team here.” (#1, FM consultant).*

*Inter/Intradisciplinary communication:* Effective communication within FM teams and across specialties was identified as critical for ensuring coordinated, patient-centred care. One interviewee emphasized the importance of flagging specific patient needs upon referral to ensure these are anticipated and addressed: “*I think it is important that people who may have issues with receiving or understanding information are flagged [to doctors] before the consultation. It should be made clear that they may need this extra time [during consultations]*” (#3, FM consultant). Participants also advocated for joint consultations involving FM specialists and other professionals, to provide consistent and comprehensive information to patients.

#### 4.2 Communication training.

*The need for communication training.* Participants unanimously advocated for structured, regular communication training for HCPs working in FM as part of their continuous professional development. Some of the participants appeared unprepared to deal with women with MHC: “*So, if I know from medical history that the patient has a diagnosis of schizophrenia or severe depression with suicidal attempts, I feel very uncomfortable to touch this topic”* (#18, FM fellow). Training focused specifically on communicating with patients with MHC was seen as essential for equipping HCPs with the skills to handle complex conversations more confidently, which could also support their own emotional resilience. As another participant expressed*, “Especially in this kind of job, I think it is very important; I would like to have specific training in this type of counseling” (#8, FM fellow).*

#### 4.3 Human factors.

*Emotional impact, coping mechanisms and compassion fatigue*. The emotional burden of delivering difficult news and witnessing patient distress, was recognized as a significant challenge for HCPs, regardless of their experience or expertise. Those with personal histories of trauma may find this particularly difficult. Historically, a lack of adequate support systems that may exacerbate these challenges has been described; in particular, one participant stated: *“…I did experience post-traumatic stress disorder probably 10 years ago now and there was no support. I wouldn’t wish it to anybody, but the fact that we’re talking about it now is a start…” (# 9, FM consultant).* While services may have improved since then, this highlights *the ongoing* need for more robust mental health resources for HCPs. When asked about coping strategies, participants mentioned consistently peer support as fundamental in managing the emotional strain of their roles. One interviewee remarked: *“… [My coping mechanism is] just speaking with colleagues; having that shared bad experience, knowing that it’s not just you….” (# 3, FM consultant).* However, participants also acknowledged that prolonged exposure to distressing situations can lead to desensitization. They urged institutions to recognise the emotional burden of delivering unexpected news and to address this by providing formal debriefing opportunities and integrating emotional and mental health support for HCPs working in FM.

### 5. The impact of health inequalities in Fetal Medicine

Interviewees identified several barriers to effective communication, many of which were linked to health inequalities. Recognizing and addressing these inequalities is essential to providing equitable care for all patients (Table 5 – S2 File).

*Healthcare resources:* Participants highlighted that resource constraints, particularly in the public sector, significantly affect the quality of care. Time constraints were a key challenge, impacting the depth and quality of interactions between HCPs and patients. As one interviewee noted: “*Time is a limit in our system; if you had all the time, you might talk for an hour and a half because there’s so much interaction, whereas we are driven to deliver most of it within 45 minutes*” (#20, neonatal consultant). Additionally, the lack of private spaces for sensitive discussions further complicates communication, as consultations may inadvertently take place in unsuitable environments.

*Language barriers*: HCPs emphasized that the limited availability of professional face-to-face interpreters hinders effective communication with non-English-speaking patients. Over-the-phone interpreting was seen as impersonal and inadequate for sensitive conversations. Many HCPs expressed concerns about the common practice of relying on family members as translators, as this can introduce biases, especially when discussing ethically complex topics. However, they admitted that at times this is the only option. One participant remarked: *“If you ask me whether I use a language line for people who can’t speak English, I will say I do. But in day-to-day life, if you call and they can’t provide an interpreter, and the husband is sitting there, you use him” (#1, FM consultant).*

*Poor health literacy:* Participants noted that low health literacy among patients pose significant barriers to effective communication. Even those with higher education can struggle to grasp complex medical information, making it even more challenging for patients with minimal education. One interviewee explained: *“Many people have no concept about the human body and how it works. They’ve no background… if you actually have to make sure that they understand every single thing you are saying on the day it’s challenging” (#9, FM consultant).* However, interviewees also warned that oversimplifying information to accommodate low health literacy may prevent patients from fully understanding the severity of their conditions.

*Mental health access:* Interviewees highlighted significant barriers to accessing mental health support, particularly for those with mild MHC. Stigma, fear of social services involvement, and underdiagnosis often prevent individuals from seeking specialist care. Even when referrals are made, long waiting times and strict eligibility thresholds further limit access. One participant explained: *“…the problem that we face is that often they don’t meet the threshold for the local area to provide that mental health support or they can be put on a waiting list that’s six months long. They’re already 3 months pregnant. They won’t be pregnant by the time they get seen…” (#16 fetal cardiology specialist nurse).* Participants noted that women with severe MHC tend to receive better support due to access to secondary mental health services, whereas those with milder conditions, who represent a much larger group, are at significant risk of deterioration due to lack of specialized care.

## Discussion

### Main findings

To our knowledge, this is the first study to explore the experiences and perspectives of HCPs working in FM on communicating unexpected news, with a specific focus on pregnant people living with MHC. Our findings highlight significant gaps in training, communication strategies, and support systems, particularly for people with mild to moderate, undiagnosed or concealed MHC, who may be at increased risk of mental health deterioration following distressing news.

Given the nature of the news typically delivered in fetal medicine, participants emphasized that all patients—regardless of known mental health history—should be treated with sensitivity and care to minimize distress and reduce the potential for trauma. They also stressed the importance of routinely checking emotional wellbeing, as many women may not disclose previous mental health conditions and may experience worsening or new-onset difficulties during pregnancy.

Equally, another significant finding of our study is the urgent need to enhance access to mental health services for those women that are not already under the perinatal mental health services.

For pregnant people with moderate/severe MHC, our HCPs highlighted the importance of adopting an MDT approach and involving an appropriate support network. This suggestion is supported by a recent umbrella review on shared decision making in mental health models [23]

Most HCPs in our study reported receiving little to no formal training in communication, relying instead on personal experience. However, all participants advocated for improved training, particularly when communicating with people with MHC.

Previous literature suggests that communication training in prenatal setting can not only enhance HCPs’ confidence and reduce anxiety in delivering difficult new [35], but also improve patients’ outcomes and experiences [36]. When it comes to exploring the communication needs of people with MHC, research has largely focused on delivering a mental health diagnosis, such as schizophrenia [24–26], rather than discussing other distressing health news. A Cochrane review highlighted a lack of high-quality evidence on the effectiveness of communication training in the context of mental health, identifying only one robust study that demonstrated improved patient experience following targeted training for healthcare professionals communicating with individuals with MHCs. The review emphasised the need for further research to assess the impact of such training [37]. Similarly, a recent Danish non-randomised intervention study reported improved patient mental health and satisfaction after interprofessional training with mental health specialists [38]. These insights along with our findings suggest that structured communication training in FM is currently inadequate and that a focus on mental health may further improve patients’ outcomes.

HCPs described several communication strategies aligning with generic medical literature on delivering unexpected news, including optimizing the setting, giving a warning shot, avoiding ambiguity, fostering understanding, and involving significant others [15,19,39]. However, novel recommendations also emerged, such as using open-ended questions to explore emotional well-being and providing ongoing support for patients after the diagnosis. Language barriers were consistently highlighted as a major challenge. HCPs expressed a preference for professional face-to-face interpreters over telephone-based services, as nonverbal cues, such as facial expressions and pauses, play a crucial role in emotionally sensitive conversations. While previous studies suggest that no interpreting mode is superior when medically trained professional interpreters are used [40–42], limited research exists on interpreting services in the context of delivering unexpected news in FM, particularly to individuals with MHC. Additionally, clinicians voiced concerns about the frequent reliance on family members as interpreters, citing risks such as selective translation and personal bias, particularly in ethically complex discussions.

Low pregnancy-related health literacy, strongly linked to socio-economic deprivation, has been long recognised as a barrier to accessing care [43] and effective communication [18,44]. Participants emphasised that in FM, low health literacy can directly affect informed consent [18], as individuals with limited understanding of medical information may decline certain investigations due to misunderstanding about their purpose or benefits. The barriers described by participants align with Nutbeam’s hierarchical model of health literacy, ranging from functional challenges (e.g., understanding terminology) to interactive difficulties (e.g., asking questions in emotionally intense situations), and critical literacy limitations, where distress may hinder a patient’s ability to appraise information and engage in shared decision-making [45].

Peer support programmes have shown promise in enhancing patient engagement and satisfaction in people with prenatal diagnosis of congenital anomalies [46,47]. Similarly, group-based antenatal education has been associated with improved psychosocial outcomes, compared to traditional individual education [48]. However, a recent review found that the benefits of community-based peer support for women with poor perinatal mental health varied depending on personal and social contexts [49]. Further research is needed to determine whether these models could enhance communication and support for pregnant individuals with MHC in FM settings.

When asked how they addressed patients’ mental wellbeing before and after delivering difficult news, many HCPs admitted reluctance to explore these issues, despite recognizing their importance. Some cited uncertainty about how to approach such conversations, while others noted that stigma surrounding MHC discourage patients from openly discussing their struggles. This aligns with prior qualitative research identifying time constraints, appointment format, and lack of training as key barriers to discussing mental health topics in FM consultations [19].

Notably, midwives and specialist nurses reported greater confidence in addressing mental health concerns. This may be attributed not only to the traditionally supportive nature of their roles but also to differences in training, with some receiving more focused education or clinical exposure to perinatal mental health. These findings highlight the importance of delivering communication training across the MDT, not only to account for different professional training backgrounds, but also to promote cohesive working practices and clarify roles and responsibilities when supporting patients.

Finally, “compassion fatigue” and burnout were recognized as significant but often overlooked issues among HCPs. Participants shared experiences of emotional distress and, in some cases, symptoms of post-traumatic stress disorder due to repeated exposure to delivering distressing news. While peer support emerged as a crucial coping mechanism to maintain empathy, there was a notable lack of formal debriefing structures or mental health resources available for FM clinicians. These findings mirror the experiences described in the context of perinatal loss, which has been explored in several studies. A recent systematic review of HCPs’ experiences in communicating such devastating news highlighted the emotional burden, uncertainty around language use, and the need for institutional and peer support [13]. This body of work reinforces the themes emerging in FM more broadly and underscores the urgent need for targeted training and systemic support, including easier access to counselling services, to sustain the emotional well-being of clinicians tasked with delivering emotionally complex news.

### Strengths and weaknesses

A key strength of this study is its novel design, as no previous research has specifically focused on this topic. Additionally, the inclusion of FM experts in the analysis helped recognize subtle nuances in the discourse that may have otherwise been overlooked. The diversity of both the HCPs interviewed and the patient populations they serve also provided a broad perspective on the experiences and challenges of delivering unexpected news.

However, some limitations must be acknowledged. Communication needs are likely to vary across the broad spectrum of mental health conditions. While our study explored HCPs’ experiences of delivering unexpected news to individuals with MHC, their suggestions may not be generalisable, as participants consistently reported a lack of training in communication relating to mental health. By exploring the perspectives of service users with MHC who have lived experience of receiving unexpected news during pregnancy, a more rounded understanding can be developed—one that enables the generation of more valid and inclusive recommendations. Such insights could form the foundation of a framework for delivering unexpected news to women with MHC and inform the development of relevant training for clinicians.

Furthermore, the study was conducted in East London, a socioeconomically deprived area where health literacy levels are often lower. HCP experiences in delivering difficult news may differ in more affluent regions, where patients generally have better health literacy. While this may limit generalizability, it also sets the foundation for improving communication in deprived areas, which contributes to equity in delivering care.

## Conclusion and future directions

This study has explored the perspectives of HCPs working within a FM MDT regarding the delivery of unexpected news to pregnant people with MHC in a multi-ethnic and socioeconomically deprived area of London. The interviewees unanimously emphasized the need for communication training and for provision of enhanced support to those receiving and delivering unexpected news.

Studies exploring patients’ experiences of receiving unexpected news during pregnancy will provide critical insights for a more patient-centred approach. Co-production of communication frameworks with HCPs and individuals with lived experience could serve as the foundation for targeted training programs in FM, ultimately improving both clinical practice and patients’ outcomes.

## Supporting information

S1 File The UNDERSTAND study protocol. This section contains the full protocol for the UNDERSTAND study, outlining the study design, methodology, and analysis.
(DOCX)

S2 File This section contains Tables 1–5, each corresponding to a distinct theme identified in the analysis. For each theme and subtheme, key recommendations are presented alongside illustrative quotes from participants to support and contextualise the findings.
(DOCX)
