# Supplementary material for: Communicating unexpected news to pregnant people living with mental health conditions in fetal medicine (the UNDERSTAND study): Healthcare professionals’ perspectives
Source: PLoS One. 2026 May 15;21(5):e0347547. doi: 10.1371/journal.pone.0347547 (PMC13178918; doi:10.1371/journal.pone.0347547)
Supplement: S1 File — This section contains the full protocol for the UNDERSTAND study, outlining the study design, methodology, and analysis. (DOCX) [file pone.0347547.s001.docx]

# Supporting Information 1 – S1.

The Understand study protocol

**Joint Research Management Office (JRMO) Research Protocol for Research Studies**

**Full Title**

**Un**expected news in pregnancy: a co-**de**sign **st**udy between service users **and** healthcare professionals to improve communication and decision-making in women with mental health conditions (UNDERSTAND)

**Short Title** UNDERSTAND

**Sponsor**  Barts Health NHS Trust (Barts Health)

Contact person:

Dr Mays Jawad

Research & Development Governance Manager

Joint Research Management Office

Dept W, Research Services

69-89 Mile End Road

London, E1 4UJ

Phone: 020 7882 7275/6574

Email: research.governance@qmul.ac.uk

**IRAS Number 310337**

***Integrated Research Application System***

**Sponsor (EDGE) Number 151171**

**REC Reference** 23/IEC08/0010

***Research Ethics Committee***

**Chief Investigator (CI)** Dr Elena Greco

Barts Health NHS Trust Senior clinical lecturer

Royal London Hospital

Fetal Medicine Unit – 8E

elena.greco@nhs.net

**List of sites** Barts Health NHS Trust

Royal London Hospital

Whipps Cross University Hospital

Newham General Hospital

PI: Elena Greco

# **1.** **Contents**

1. Contents

2. Glossary

3. Summary and synopsis

4. Introduction

4.1. Background

4.2. Rationale

5. Study objectives

5.1. Primary objective

5.2. Secondary objective

5.3. Primary endpoint

5.4. Secondary endpoint

6. Study population

6.1. Inclusion criteria

6.2. Exclusion criteria

7. Study design

21. References

#

# **2.** **Glossary**

Antenatal Results and Choices - ARC

Fetal Medicine Services - FMS

Healthcare Professionals - HCPs

Mental Health Conditions - MHC

Patient and Public Involvement - PPI

Work Package – WP

# **3.** **Summary and synopsis**

| **Short title** | UNDERSTAND |
| --- | --- |
| **Methodology** | Qualitative study |
| **Objectives / aims** | The overarching aim of this study is to co-produce with service users and HCPs recommendations to feed into a comprehensive framework to improve delivery of unexpected news in patients with MHC. We will achieve this by fulfilling the objectives below:  *Objective 1:* To identify and understand barriers and facilitators during fetal medicine consultations with patients with MHC (WP1)  *Objective 2:* To explore the lived experience of women with MHC and that of their significant others, with regards to the way they have received unexpected news in pregnancy from HCPs (WP2)  *Objective 3:* To explore the practices, experiences and challenges of fetal medicine HCPs on delivering unexpected news to pregnant patients with MHC (WP3)  *Objective 4:* To Co-design recommendations for improvement and developing a framework (WP4) |
| **Number of participants** | 90 |
| **Inclusion and exclusion criteria** | **WP1-2**  Active diagnosis of anxiety, depression, bipolar affective disorder (BAD), post-traumatic stress disorder (PTSD) and psychotic disorders,  **AND**  referral to fetal medicine Unit for ongoing (WP1) or past (WP2) pregnancy complications (e.g. structural defect, chromosomal or genetic condition, high chance of chromosomal syndrome at combined prenatal screening, intrauterine growth restriction, intrauterine death, abnormal placentation, multiple pregnancies)  **AND**  Age > 16 years  Capacity to consent to taking part in the study  **AND/OR**  Patients’ significant others, can be recruited together or instead of patients upon patients’ preferences  *WP1: Patients with severe mental health problems are case loaded by our specialist midwives. For these patients we will consult their specialist midwife, before approaching the patient, on the suitability of the patient to take part in the study.  *Patients for WP2 will be considered suitable for recruitment 6 months after a previous pregnancy complicated by unexpected news and 12 months after a pregnancy loss or bereavement.  **WP1-3**  Consultants or senior clinical fellows in fetal medicine, Neonatal Medicine, Fetal Cardiology, Clinical genetics, Paediatric surgery  Specialist fetal medicine midwives, specialist fetal cardiac nurses  Antenatal Screening, Bereavement, Maternity Safeguarding midwives  Obstetric Sonographers  **at least 1-year experience in the profession*  **WP4**  All participants from WP2,3 |
| **Statistical methodology and analysis (if applicable)** | WP1: Conversation analysis  WP2, 3: Thematic analysis  WP4: No analysis |
| **Study duration** | 24 months (End date: 03/02/2025) |

# **4.** **Introduction**

This is a co-production project between researchers, healthcare professionals (HCPs) and service users, utilising qualitative methods. This study was developed as a response to the voices of many of our women who felt unheard, misunderstood and judged when they had to make difficult decisions about their pregnancies. Those women are now calling for improvement in discussing difficult news and, to be given equal opportunities to exercise their rights and choices regarding their health and their pregnancy.

The idea of this project originated from members of Katie’s Team, our East London women’s health research advisory group, who suggested that women from diverse backgrounds with mental health conditions (MHC) in these situations could be better supported with their decision-making. The idea to co-produce this research was put forward by the group and agreed with the researchers.

The study will be carried out in collaboration with Antenatal Results and Choices (ARC), of which our PPI lead (JF) is the director of. This is the only national charity supporting expectant parents who face difficult decisions about antenatal tests and results; ARC also offers training to help HCPs provide the right care to every parent with unexpected prenatal diagnosis.

The study will be guided and supported by a Steering Committee including HCPs from relevant disciplines, experts in counselling and supporting women during pregnancies, women with MHC with a history of receiving unexpected news during pregnancy and their significant others.

Finally, in the present protocol, we have referred to pregnant persons as ‘women’. However, during the recruitment and implementation of the project, the research team will ensure that the preferred gender identity of the participants is recorded and used accordingly.

## **4.1.** **Background**

Advances in prenatal screening and clinical genetics over the last decades have facilitated early diagnosis of several structural and genetic fetal conditions. A significant fetal structural or genetic anomaly is detected in about 2-3% of pregnancies^1^. These findings can vary from conditions that have smaller effects on the baby and can be potentially surgically corrected, such as cleft lip/palate, to serious conditions that can lead to lifelong complications, such as spina bifida and complex congenital heart diseases. Occasionally some conditions, like anencephaly, will be life-limiting. Other referrals to fetal medicine include more common findings, albeit equally unexpected for the parents, such as high chances of genetic condition at prenatal screening or growth restriction. Overall, one in 5 pregnant women would have to discuss with a fetal medicine specialist the possibility of a fetal condition and its management options which may include, in serious cases, having to decide about the future of the pregnancy^2^.

Delivering unexpected news regarding the unborn baby may be a source of significant psychological distress both for expectant parents and healthcare professionals (HPCs). Anxiety, depression, post-traumatic stress and difficulties in parental bonding have been reported during and after pregnancies in couples with complex prenatal diagnoses, irrespective of the outcomes^3,4^. Poor maternal psychological wellbeing may increase further the risk of unfavourable pregnancy outcomes including impaired fetal growth and prematurity^5^. Delivering unexpected news may also cause distress, guilt and anger to the person disclosing it, often due to lack of training, fear of the patients’ emotional response or inability to detach from a previous personal experience ^6^.

In pregnant women with an underlying mental health condition (MHC), discussing unexpected news may be immensely challenging. Mental illnesses as well as psychiatric medications are known to impact receptive and expressive language, speech and social communication^7^ thus potentially making *any* interaction between patients and HCPs more difficult. HCPs, on the other hand, have the tendency to shroud the information, focussing on the medical details^8^ or using mitigating language and avoiding clarification of the diagnosis in an attempt to prevent uncontained emotional responses^9^.

*Preliminary data*

Twenty percent of women in the perinatal period - from conception to 12 months after delivery- will experience a MHC, most commonly depression^10^. Psychiatric disorders with onset before or during pregnancy are known to have a significant impact on maternal morbidity and mortality^11^. That 1 in 5 pregnant women have MHC^12^, along with the paucity of research or guidance on how HCPs should deliver unexpected news to them warrant the need to prioritise research in this area. Evidence-based recommendations on how to manage these difficult conversations in turn will promote equity in the delivery of maternity care and enhance informed decision making for underserved women.

Data on our local population, an ethnically diverse community in East London, suggest that ~30% of women at booking report a history of MHC. This means that out of the total 17.000 women booked for their pregnancies with Barts Health every year, approximately 3400 will require specialist fetal medicine assessment. Of those, over one third will have a MHC impacting communication.

We carried out a survey on 50 consecutive pregnant women followed up within our fetal medicine Unit for various fetal conditions including impaired fetal growth and structural defects, to assess maternal psychological well-being after receiving unexpected news. We repeated the 4 questions (Whooley and GAD-4^13^) used at booking for screening for MHC following their interaction with our fetal medicine Unit. The wellbeing score deteriorated in 63% of mothers with known MHC and only in 8% of those with no known or suspected MHC after receiving unexpected news.The results suggested that patients with MHC are psychologically more affected by unexpected news in pregnancy than other parents.

We conducted a further survey to explore fetal medicine HCPs’ perspectives on the need for guidance on how to communicate unexpected news. Seventy-one HCPs working in the UK filled the questionnaires, 63% of whom deliver unexpected news more than once weekly. Interestingly, 40% of the responders have never received any formal training on delivering unexpected news and 20% have had training once. The majority (84%) of HCPs agreed that training on delivering unexpected news tailored to HCPs working in the fetal medicine field would be useful, particularly when dealing with specific groups of women, including women with MHC.

*Knowledge gap*

We conducted a systematic search of the literature and identified several tools to support best practice in delivering unexpected news during pregnancy, aiming to increase clinicians’ confidence, reduce stress and facilitate patient involvement in decision-making ^14,15,16^. However, these strategies lack robust supporting evidence; they are designed “by doctors for doctors'' rather than co-produced with service users' input focusing on patients’ specific needs and outcomes. Therefore, none of these models fully meet the needs of patients with MHC or truly reflect the clinical experience of HCPs and further guidance is warranted to improve clinical practice.

Practical recommendations for optimising communication between mental health clinicians/allied professionals and patients with MHC also exist in literature^7^. However, the outcomes of psychiatric research conducted on the general population may be difficult to apply to pregnant women. Adaptation to pregnancy may present unique challenges as expectant women with a MHC may have impaired ability to internalise information. Research in communicating news to people with MHC is mostly quantitative^17^. In-depth qualitative analysis of communication with people with MHC has been limited to psychotherapy and mental health settings ^1819,20^, thus neglecting communication about physical health topics.

## **4.2.** **Rationale**

This study will bring fetal medicine HCPs and service users (patients with MHC with their significant others) to work together to highlight barriers and facilitators of difficult conversations and agree on recommendations for improving communication and decision-making. These recommendations will enable us to develop a comprehensive framework on how to disclose unexpected news during pregnancy to patients with MHC.

Effective dissemination of our framework will promote equity and inclusivity in prenatal care by giving equal rights to all women to understand their health conditions and to make decisions about their pregnancy.

Furthermore, by improving the psychological wellbeing of service users this research is expected to have a positive impact on pregnancy outcomes and patients’ satisfaction. Similarly, by reducing stress of HCPs, this research can contribute to staff wellbeing and retention.

Finally, coproduction is an innovative yet proven research approach where service users and HCPs work alongside each other to identify problems that can be practically overcome and to develop implementable solutions that benefit everyone. This approach was overwhelmingly supported by members of Katie’s team (East London Patient and Public engagement team). The output of this study has the potential to substantially improve communication, make a difference to the experience of underserved patients and empower them to make decisions for their pregnancies.

# **5.** **Study objectives**

## **5.1.** **Primary objectives**

*Objective 1:* To identify and understand barriers and facilitators during fetal medicine consultations with patients with MHC (WP1)

*Objective 2:* To explore the lived experience of women with MHC and that of their significant others, with regards to the way they have received unexpected news in pregnancy from HCPs (WP2)

*Objective 3:* To explore the practices, experiences and challenges of fetal medicine HCPs on delivering unexpected news to pregnant patients with MHC (WP3)

*Objective 4:* To co-design with service users and HCPs recommendations to develop a comprehensive framework for improving the delivery of unexpected news and decision-making during pregnancy.

## **5.2.** **Secondary objective**

N/a

## **5.3.** **Primary endpoint**

To co-develop a framework for effective communication when delivering unexpected news in pregnant women with MHC

## **5.4.** **Secondary endpoint**

N/a

# **6.** **Study population**

The project will be conducted across the three maternity units within Barts Health NHS Trust (Royal London, Whipps Cross, Newham), the largest NHS Trust in England, with approximately 17,000 deliveries/ year. Barts Health serves a socially deprived, diverse population with over 70% of women being non-white and with one of the highest rates of consanguinity in the UK which in turn, increases further the risk of unexpected fetal diagnosis.

The fetal medicine services (FMS) at Barts Health include three multi-disciplinary teams based at the three maternity units which work in a collaborative manner. Each team includes 20 to 30 staff members (see inclusion criteria). The FMS operate as an emergency arm of the maternity unit and are co-located with other maternity services (antenatal clinic, obstetric triage, delivery suite and screening services) from which referrals for suspected or confirmed fetal conditions are received.

Barts Health FMS receive over 3,000 referrals/year, with an average of over 60/week; the referrals are processed within 24 hours and women are invited to attend an appointment within 3 days from the referral. Waiting times on the day of the appointment range between 15 to 90 minutes, depending on workload and case complexity.

Our local data suggest that every year about 900 pregnant patients with MHC are referred to our Fetal Medicine Units, with most of them requiring multiple counselling and follow-up sessions.

The study comprises four work packages.

**WP1:**

We will obtain consent for filming prenatal consultations between Fetal Medicine HCPs working within the three Fetal Medicine Teams at Barts Health and patients with MHC and their significant others.

*Service users’ screening/recruitment:* Patients screening will be carried out from the hospital electronic booking system by a member of the research team who is also part of the direct clinical team, ahead of patients’ clinical visits.

Patients eligible for recruitment (**section 7.1**) will be approached on the day of their appointment with HCPs by a member of the clinical team who will explain the study in detail and provide the patient information sheet. A written consent will be obtained prior to the procedure by a researcher with the help of language advocates, as appropriate.

Recruiting patients from the ethnically heterogeneous population across the whole Trust, will further amplify ethnic, socio-cultural and religious diversity. See **section 9** for further information on recruitment of service users.

*HCPs screening/recruitment:* HCPs working within the three multi-disciplinary FMS at Barts Health (**section 7.1**) will be approached for recruitment. Potential participants will be invited to a group meeting (virtual and/or face to face [F2F]) where the study aims and methods will be explained in detail by the clinical research team. To those interested in taking part, the participants’ information leaflet and consent form will be provided. HCPs will have the option to consent for participation to WP1 only, or to be involved in two or all the remaining work packages involving HCPs (WP3 and WP4). Written consent will be obtained from all participants.

Given the results of our survey which highlighted the need for formal guidance on how to deliver unexpected news and from informal discussions with the clinical teams across the three sites, we anticipate that most HCPs will volunteer to participate in the study. Including HCPs working on different sites, with varied demographic characteristics, experience and roles will ensure a wider sample of HCPs.

**WP2:**

We will recruit patients with MHC with lived experience of receiving unexpected news during pregnancy and/or their significant others to share their experiences in an interview with our researchers.

Service users eligible for recruitment (**section 7.1**) to WP2 will be identified across London via media advertising and charity contacts (e.g., ARC, Mental Health charities). We will purposely recruit patients with a range of MHC, as well as from different socio-ethnic and religious backgrounds.

To those interested in taking part, the study will be explained in detail by the research team during an individual meeting (virtual and/or F2F) and participants’ information leaflet and consent form will be provided. Service users will have the option to consent for participation to WP2 only or WP2 and WP4. We will not include service users recruited for WP1 to avoid further emotional burden to patients with ongoing pregnancies complicated by fetal conditions. For WP2, verbal/written consent will be sought for women alone or together with their significant others or for their significant others only, to give an interview conducted by the researchers and for the content to be recorded, transcribed, analysed and used anonymously.

**WP3:**

We will recruit HCPs working within Barts Health FMS to share experiences and challenges when delivering unexpected news in patients with MHC through an interview with our researchers.

HCPs to be recruited in WP3 (**section 7.1**) will be identified as per WP1. Those who agree to participate in WP3 will consent to give an interview conducted by the researchers and for the content to be recorded, transcribed, analysed and used anonymously.

Diversity in roles, grades and sites is important to ensure representation of everyone involved in the patient's journey from the initial contact until the decision-making.

**WP4:**

Individuals recruited for WP2 and WP3 will be asked to consent to participate also in the joint workshop. In case of drop-outs, new participants will be identified, recruited, and consented using the same methods. Participants in WP4 will consent to take part and be recorded in a group workshop.

## **6.1.** **Inclusion criteria**

**Inclusion criteria for service users in WP1**

Ongoing pregnancy - any gestational age

AND

Age > 16 years

Capacity to consent to taking part in the study

AND

Active diagnosis of anxiety, depression, bipolar affective disorder (BAD), post-traumatic stress disorder (PTSD) and psychotic disorders,

AND

referral to fetal medicine Unit for ongoing pregnancy complications (e.g. structural defect, chromosomal or genetic condition, high chance of chromosomal syndrome at combined prenatal screening, intrauterine growth restriction, intrauterine death, abnormal placentation, multiple pregnancies)

AND/OR

Patients’ significant others (any age, any gender, any relationship), can be recruited together or instead of patients upon patients’ preferences

*Patients with severe mental health problems are caseloaded by our specialist midwives. For these patients we will consult their specialist midwife, before approaching the patient, on the suitability of the patient to take part in the study.

**Inclusion criteria for service users in WP2**

Age > 16 years

Capacity to consent to taking part in the study

AND

History of previous pregnancy with complications requiring referral to a fetal medicine unit. (e.g. structural defect, chromosomal or genetic condition, high chance of chromosomal syndrome at combined prenatal screening, intrauterine growth restriction, intrauterine death, abnormal placentation, multiple pregnancies)

AND

Concurrent diagnosis of anxiety, depression, bipolar affective disorder (BAD), post-traumatic stress disorder (PTSD) and psychotic disorders,

AND/OR

Patients’ significant others (any age, any gender, any relationship), can be recruited together or instead of patients upon patients’ preferences

*Patients for WP2 will be considered suitable for recruitment 6 months after a previous pregnancy complicated by unexpected news and 12 months after a pregnancy loss or bereavement.

**Inclusion criteria for Healthcare professionals to be recruited in WP1, WP3**

Consultants or senior clinical fellows in fetal medicine, Neonatal Medicine, Fetal Cardiology, Clinical genetics, Paediatric surgery

Specialist fetal medicine midwives, specialist fetal cardiac nurses

Antenatal Screening, Bereavement, Maternity Safeguarding midwives

Obstetric Sonographers

AND

Any gender

No age limits

AND

At least 1-year experience in the profession

**Inclusion criteria for Service Users and Healthcare professionals to be recruited in WP4**

As per WP2 and WP3.

## **6.2.** **Exclusion criteria**

**Exclusion criteria WP1**

Women less than 16 years of age

Women with a past (not ongoing/resolved) diagnosis of mental health disorders

Women with a safeguarding order in place

Women that lack the capacity to give consent.

HCPs who do not consent to their consultations being recorded or with less than one year experience in the profession.

**Exclusion criteria WP2**

Women less than 16 years of age

Women with a safeguarding order in place

Women that lack the capacity to give consent.

Women without a past history of pregnancy complications requiring referral to a fetal medicine unit

Women without a previous concurrent history of mental health disorders {anxiety, depression, bipolar affective disorder (BAD), post-traumatic stress disorder (PTSD) and psychotic disorders}

**Exclusion criteria WP3**

HCPs with less than one year of experience in the profession or not willing to consent

**Exclusion criteria WP4**

As per WP2 and WP3

# **7.** **Study design**

**WP1**

Aims: To analyse the structure and content of prenatal consultations between fetal medicine HCPs and service users.

*Methods:*

Conversation analysis of filmed, face-to-face prenatal consultations involving HCPs from the multi-disciplinary fetal medicine teams and service users. Conversation analysis is an established method in healthcare to identify challenges and solutions faced by patients and HCPs, contributing to the development of interventions to improve practice ^22,23^. As per qualitative methodology, the sample size will be based on information power^24^, but experience of the research team estimates 20-30 recordings from a minimum of 5 to 7 HCPs should provide sufficient variability in clinician style and news content, and will be feasible within the time frame.

*Filming of consultations:* The recording equipment will be set up in a careroom, where consultations take place after the scan is completed. The research team will not be present during the scan/consultation. The main clinician conducting the consultation will turn on a small, unobtrusive camera, once all parties have consented in advance. The conversation will continue as normal and at the end the HCP will stop the recording. The researcher will download the video-recording from the camera and store it into an encrypted device, for further transcription and analysis. Previous research has shown minimal change of behaviour due to being observed^25^, hence we expect to capture practice as it occurs naturally.

*Data analysis:* Filmed consultations will be watched by the research team and key extracts related to the delivery of the unexpected news will be identified for analysis. These will be transcribed using Jeffersonian conventions for micro-analysis of verbal and non-verbal communication to provide insight into what people say (e.g. structure of the conversation, choice of words) and how they say it (e.g. body language, voice tone, speed, pauses). Service user responses will also be identified to describe how certain formats of HCP communication impact on service user experience. Findings of WP1 will be used to develop topic guides for WP2-3 and material for discussion in WP4.

An experienced conversation analyst will lead the analysis, with repeated analysis meetings with the research team and Steering Group adding transparency and hence reliability to the findings^26^.

**WP2**

Aim: To learn from the lived experience of service users, with regards to the way unexpected news were disclosed to them by HCPs.

*Methods:*

We will interview women with MHC and significant history of fetal complications during a previous pregnancy and/or their significant others separately or together, according to participants’ preferences.

*Interviews:* Interviews for WP2 will take place either F2F or virtually according to the interviewees’ preferences. Interviews will be arranged at a time that is convenient for the interviewees, and women will have the option to be interviewed alone or together with their significant others or for these to be interviewed instead of themselves. We will aim for virtual interviews to be over sponsor approved video conferencing software, but if participants have difficulty using these platforms we will conduct them over telephone. Where F2F interviews are preferred, these will be arranged at a location convenient to the interviewees.

An experienced qualitative researcher, with expertise in interviewing people about sensitive topics, will conduct these interviews rather than researchers with clinical roles, in order to maximise ease/openness of conversation and participants’ engagement. ARC staff will be present at the interview alongside the interviewer and be available for emotional support and/or further counselling, should the interviewee find the conversation distressing. The interviews will be semi-structured, following a topic guide co-designed by the research team (including PPI members) together with the Steering Committee.

The interviews will be expected to last between 30 and 45 minutes. The researcher will encourage service users to share their lived experiences, identify positive and negative aspects of the communication and suggest areas of improvement. The content of the interviews will be recorded and stored into a secure, bespoke electronic study database for transcription and analysis anonymously .

*Data-analysis:* We will analyse material in parallel with data collection, so that the sample could be lower/higher depending on information power. Based on experience, the research team estimates 20-30 interviews should suffice. The conversations will be audio-recorded and transcribed verbatim. Reflexive thematic analysis of the interviews will allow identification of shared views between the participants and the development of themes^27^. Relevant themes will then be categorised into key areas of improvement, with regular referral to the data to ensure accurate representations of the participants’ views and experiences. Findings from the analysis will be shared with service users and HCPs in WP4. An experienced qualitative researcher will conduct the analysis, with support and double coding from other members of the research team with experience in qualitative studies.

**WP3**

Aims: To learn from the experience of fetal medicine HCPs, with regards to the way they disclose unexpected news to patients with MHC.

*Methods:*

*Interviews:* Twenty to thirty interviews with HCPs. Diversity in roles, grades and sites is important to ensure representation of everyone involved in the patient's journey from the initial contact until the decision making.

An experienced qualitative researcher, with expertise in interviewing people about sensitive topics, will conduct these interviews rather than researchers with clinical roles, in order to minimise the risk of HCPs feeling challenged/judged by their colleagues. Type (F2F versus virtual), time and location of the interview will be agreed upon interviewees’ preferences. The interview will follow a topic guide, co-designed by the Steering Committee and PPI co-applicants, with the aim to explore HCPs experiences and identify potential barriers and enablers to communication, while pointing out areas of improvement. Should the interviewee find the conversation distressing, the interviewer will signpost sources of psychosocial support either directly or through the Trust relevant care pathways.

*Data analysis:* The conversations will be audio-recorded and transcribed verbatim. The same analysis methods will be used from WP2 and the findings will be shared in WP4.

**WP4**

To co-design with service users and HCPs recommendations to develop a comprehensive framework for improving the delivery of unexpected news and decision-making during pregnancy.

*Methods:*

Workshop of 20-30 participants including HCPs and service users from WP2 and WP3, and facilitated by the research team, including members with clinical roles and PPI. Bringing HCPs and service users together to co-design recommendations and solutions for improvements is an innovative yet already proven resource^28^.

*Workshop:* We will ensure diverse representation both from service users (socio-cultural background, type of MHC) and HCPs (gender, age, role, cultural background).

The workshop will be led by two members of the research team with appropriate training and experience in conducting workshops and focus groups. Members of the research team including researchers, clinicians, and public members will pair up (one clinician/researcher and one public member) to co-moderate small group discussions (3-4 groups of 5-10 participants). The workshop will be mainly virtual to minimise geographical inconvenience and optimise candour and confidentiality. However, multiple options for participation will be available to safeguard anonymity and address possible digital inequalities (see section 9). The workshop will be recorded to ensure that all recommendations are captured.

Themes emerged from WP1, WP2 and WP3 will be shared at the beginning of the meeting with all participants. The attendees will then be assigned to heterogeneous break-out rooms (focus groups), where structured discussion will follow, led by members of the research team. Each break-out room will focus on 1 to 3 specific themes identified from WP1-WP3 (e.g., Who, When, Where etc.. ) using 3 to 6 open questions (e.g.Who? Who should be leading the consultation? etc) and allowing 5-10 minutes per question. A final whole group discussion will summarise recommendations from the break-out rooms and draft a framework for delivering unexpected news in pregnancy, which will be finalised by the research team.

*Framework:* The recommendations from WP4 will be organically arranged in a co-designed and co-produced framework which will truly reflect clinical reality, patients’ preferences and professional needs. The framework will be drafted during the workshop meeting and further elaborated by the research team prior to sharing it with participants, patients and the public.

# **8.** **References**

1. Cunningham FG, Leveno KJ, Bloom SL. Williams Obstetrics. (24th edition). New York; USA: McGraw-Hill Education; 2014;. p.:283.

2. Luz, R., George, A., Spitz, E., & Vieux, R. (2017). Breaking bad news in prenatal medicine: a literature review. Journal of reproductive and infant psychology, 35(1), 14–31. https://doi.org/10.1080/02646838.2016.1253052

3. Goecke, T. W., Voigt, F., Faschingbauer, F., Spangler, G., Beckmann, M. W., & Beetz, A. (2012). The association of prenatal attachment and perinatal factors with pre- and postpartum depression in first-time mothers. Archives of gynecology and obstetrics, 286(2), 309–316. https://doi.org/10.1007/s00404-012-2286-6

4. Beauquier-Maccotta, B., Chalouhi, G. E., Picquet, A. L., Carrier, A., Bussières, L., Golse, B., & Ville, Y. (2016). Impact of Monochorionicity and Twin to Twin Transfusion Syndrome on Prenatal Attachment, Post Traumatic Stress Disorder, Anxiety and Depressive Symptoms. PloS one, 11(1), e0145649. https://doi.org/10.1371/journal.pone.0145649

5. Rondó, P. H., Ferreira, R. F., Nogueira, F., Ribeiro, M. C., Lobert, H., & Artes, R. (2003). Maternal psychological stress and distress as predictors of low birth weight, prematurity and intrauterine growth retardation. European journal of clinical nutrition, 57(2), 266–272. https://doi.org/10.1038/sj.ejcn.1601526

6. Oliveira, F. F., Benute, G., Gibelli, M., Nascimento, N. B., Barbosa, T., Bolibio, R., Jesus, R., Gaiolla, P., Setubal, M., Gomes, A. L., Francisco, R. P., & Bernardes, L. S. (2020). Breaking Bad News: A Study on Formal Training in a High-Risk Obstetrics Setting. Palliative medicine reports, 1(1), 50–57. https://doi.org/10.1089/pmr.2020.0014

7. Rees H, Forrest C, Rees G. Assessing and managing communication needs in people with serious mental illness. 2018 Nursing Standard. doi: 10.7748/ ns.2018.e11104

8. Maynard, Douglas & Frankel, Richard. (2006). On diagnostic rationality: Bad news, good news, and the symptom residue. 10.1017/CBO9780511607172.011.

9. Karnieli-Miller O, Werner P, Aharon-Peretz J, Eidelman S. Dilemmas in the (un)veiling of the diagnosis of Alzheimer's disease: walking an ethical and professional tight rope. Patient Educ Couns. 2007 Aug;67(3):307-14. doi: 10.1016/j.pec.2007.03.014. Epub 2007 Apr 20. PMID: 17449215.

10. https://www.who.int/teams/mental-health-and-substance-use/maternal-mental-health

11. Knight M, Bunch K, Tuffnell D, Shakespeare J, Kotnis R, Kenyon S, Kurinczuk JJ (Eds.) on behalf of MBRRACE-UK. Saving Lives, Improving Mothers’ Care - Lessons learned to inform maternity care from the UK and Ireland Confidential Enquiries into Maternal Deaths and Morbidity 2016-18. Oxford: National Perinatal Epidemiology Unit, University of Oxford 2020.

12. https://www.rcpsych.ac.uk/mental-health/treatments-and-wellbeing/mental-health-in-pregnancy

13. National Institute for Health and Care Excellence (2014). Antenatal and postnatal mental health: clinical management and service guidance. [Nice guideline No. 115] https://www.nice.org.uk/guidance/qs115

14. Atienza-Carrasco, J., Linares-Abad, M., Padilla-Ruiz, M., & Morales-Gil, I. M. (2018). Breaking bad news to antenatal patients with strategies to lessen the pain: a qualitative study. Reproductive health, 15(1), 11. https://doi.org/10.1186/s12978-018-0454-2

15. Guerra, F. A. R., Mirlesse, V., & Baião, A. E. R. (2011). Breaking bad news during prenatal care: A challenge to be tackled. Ciência & Saúde Coletiva, 16(5), 2361–2367.

16. Johnson, J., Arezina, J., Tomlin, L., Alt, S., Arnold, J., Bailey, S., Beety, H., Bender-Atik, R., Bryant, L., Coates, J., Collinge, S., Fishburn, J., Fisher, J., Fowler, J., Glanville, T., Hallett, J., Harley-Roberts, A., Harrison, G., Horwood, K., ... Hardicre, N. (2020). UK consensus guidelines for the delivery of unexpected news in obstetric ultrasound: The ASCKS framework. Ultrasound, 28(4), 235-245. https://doi.org/10.1177/1742271X20935911

17. Alyssa C. Milton & Barbara A. Mullan (2014) Communication of a mental health diagnosis: a systematic synthesis and narrative review, Journal of Mental Health, 23:5, 261-270, DOI: 10.3109/09638237.2014.951474

18. Dilekler, İ. (2021). Conversation Analysis in Psychotherapy Research: A Methodological Review. Studies in Psychology, 41(1), 1-34.

19. 1Dooley, J., Bass, N., & McCabe, R. (2018). How do doctors deliver a diagnosis of dementia in memory clinics? The British Journal of Psychiatry, 212(4), 239-245. doi:10.1192/bjp.2017.64

20. Joseph Ford, Felicity Thomas, Richard Byng and Rose McCabe: BJGP Open 2019; 3 (4): bjgpopen19X101670. DOI: <https://doi.org/10.3399/bjgpopen19X101670>

21. Nijjar SK, D'Amico MI, Wimalaweera NA, Cooper N, Zamora J, Khan KS. Participation in clinical trials improves outcomes in women's health: a systematic review and meta-analysis. BJOG. 2017 May;124(6):863-871. doi: 10.1111/1471-0528.14528. Epub 2017 Feb 14. PMID: 28194870

22. Rebecca K. Barnes (2019) Conversation Analysis of Communication in Medical Care: Description and Beyond, Research on Language and Social Interaction, 52:3, 300-315

23. Jeffrey D. Robinson & John Heritage (2014) Intervening With Conversation Analysis: The Case of Medicine, Research on Language and Social Interaction, 47:3, 201-218,

24. Malterud K, Siersma VD, Guassora AD. Sample Size in Qualitative Interview Studies: Guided by Information Power. Qualitative Health Research. 2016;26(13):1753-1760.

25. Goodwin S, McGuirk M, Reeve C. The impact of video telehealth consultations on professional development and patient care. Aust J Rural Health. 2017 Jun;25(3):185-186.

26. Peräkylä, A. (2011). Validity in research on naturally occurring social interaction. Qualitative research, 365, 382.

27. Virginia Braun & Victoria Clarke (2019) Reflecting on reflexive thematic analysis, Qualitative Research in Sport, Exercise and Health, 11:4, 589-597

28. Kynoch K, Ramis MA. Experience based co-design in acute healthcare services: a scoping review protocol. JBI Database System Rev Implement Rep. 2019 Jan;17(1):3-9
