# Supplementary material for: Communicating unexpected news to pregnant people living with mental health conditions in fetal medicine (the UNDERSTAND study): Healthcare professionals’ perspectives
Source: PLoS One. 2026 May 15;21(5):e0347547. doi: 10.1371/journal.pone.0347547 (PMC13178918; doi:10.1371/journal.pone.0347547)
Supplement: S2 File — For each theme and subtheme, key recommendations are presented alongside illustrative quotes from participants to support and contextualise the findings. (DOCX) [file pone.0347547.s002.docx]

# Supporting Information 2 – S2.

Tables 1-5

**Table 1. Enhancing patient understanding**

| **Key Ideas – Recommendations** | **Quotes** |
| --- | --- |
| **Optimizing setting**   - Speak with patients while dressed and comfortable and maintain eye contact; patients lying down with their abdomen exposed may feel more vulnerable. - Avoid starting the consultation while scanning, unless there is something that the parents would benefit to watch on screen - Consider moving to a quiet room, where everyone can sit in a circle for conducting the consultation | - I *like to do it when I'm not scanning them, if possible; you're more comfortable receiving news when you're dressed as opposed to half naked covered in gel. #*2, FM fellow - *If I need to show them something, often I'll show them while they're lying down on the scan.* # 3, FM consultant - *I think the light in the room is quite important and how your body language is, to make them feel more relaxed*. #12, FM midwife |
| **Clarity and Honesty**   - Begin the consultation by anticipating its difficult content with phrases such as “I am sorry, I don’t have good news for you today” (“*Warning shot*”) - Be clear regarding severity of condition and possible outcomes for the baby. - Be honest regarding uncertainty of findings Avoid medical jargon and statistics, as most patients would not understand. | - *I'd like to start off by probably giving like a warning shot and say, you know, I've scanned your baby today. There is something that I'm worried about. #2, FM fellow* - *I can talk about statistics, but unless you specifically say, look, your baby will need tricky operations and may not survive the operations, then they don't get it. #20, Neonatal consultant* - *I don't want to hide anything regarding the severity of the problem. So, with or without mental health problem, I think we should be just clear and honest. #8, FM* |
| **Checking understanding**   - Ask patients to summarize their understanding of the information shared - Encourage patients to read information leaflets written in accessible language - Ensure that misconceptions are addressed so that patients are able to make truly informed decisions | - “Can you please tell me, what do you think is the problem?” And then I let them tell me what they understand, because sometimes it's actually they did not understand anything. Participant 18, FM fellow - Information leaflets that are written in a way that people can understand. So even if on the day you worry that they haven’t taken the information in, giving them something written to take away with them is really important. *#*13, Fetal cardiology specialist nurse |
| **Allowing time for processing information**   - Provide information in small, manageable chunks, observing the patient's reactions, and allowing time for patients to process information before presenting more. - Consider pausing the consultation if patient appears overwhelmed and suggest continuing at a later time/ follow-up appointment | - I think it's very, very useful because then people have some time to digest information, perhaps to read more and to discuss with family what they want to do... *#* 18, FM fellow |
| **Non-directive counselling**   - Clear and compassionate communication - Remind patients that the final decision rests with them and that the HCP’s role is to support them, regardless of the decision made, without any judgment. | - …the important thing for you would be to understand what this means for your baby. So what the baby will look like, what type of problems the baby may have…. *#* 6, FM consultant |

**Table 2.1 Practical considerations for supporting patients throughout their consultations**

| **Key ideas – Recommendations** | **Quotes** |
| --- | --- |
| **Role of significant others**   - Prepare patients for the nature of information that may be discussed in subsequent consultations and encourage them to attend with someone who can provide support. - If significant others are not present at the start of the scan, enquire about anyone’s availability and consider rearranging appointment to facilitate their participation. | - *Because you as a practitioner, if you think you will be breaking news it’s important to find out if there is someone who can come with them to assist them for kind of emotional support.* #7, FM midwife` |
| **Explaining silences**   - Pre-emptively address silences by informing patients beforehand, using statements like, “I may be silent during the scan so that I can focus. Please, do not worry”. - Explain the need for senior presence in the room | - *Here's one thing that many patients say: that “the sonographer or the junior doctor abandoned the room” and so they didn't say anything, or they said “I'm going to call someone” and that for them, they tend to describe it to me as very stressful #17, FM consultant* |
| **Addressing future challenges**   - Endeavour to have joint consultations with relevant HCPs, including neonatologists, midwives, obstetricians, etc. - Provide signposting to sources of information and support - Address in simple and clear terms the practical aspects and challenges of caring for a baby with health issues as well as possible outcomes, including worst and best scenarios. | - *There are a lot of resources and support groups available depending on the kind of condition; so, we print out the information and we give them telephone numbers they can ring and get some support and information.* # 10, FM consultant |
| **Continuity of care**   - Endeavour to provide continuity of care for all patients in the fetal medicine setting, unless otherwise requested/indicated. - Maintain open communication by encouraging patients to call back if they have further questions or concerns. | *I always make it very clear that we're always here to support our women after unexpected news; they can always call us here in the office… I would say I have phone calls daily from patients wanting to discuss previous scans…*  #12, FM midwife |

**Table 2.2 Providing emotional support**

| **Key ideas – Recommendations** | **Quotes** |
| --- | --- |
| **Monitoring mental wellbeing**   - Acknowledge patients’ emotional distress during consultations - Regularly monitor patient’s emotional wellbeing after news is delivered (e.g., include open-ended questions at subsequent follow-ups "How are you coping?", obtain consent for courtesy phone calls a few days after the news has been delivered) | - *In this job, you deliver bad news every day. But how you deliver it and how you support the family after that, is really key to how they cope with it. #13, Fetal cardiology specialist nurse* - *Basically, support, ability to understand the risk for that woman thereafter. Will she go home and have a serious mental health disturbance? Is there support in place for that? #16, Fetal cardiology specialist nurse* - *This is the perfect storm really to exacerbate any of preexisting mental health concerns, even for those who are not known to have mental health concerns. #4, Neonatal consultant* |
| **MDT approach for women with known MHC**   - Involve relevant individuals, such as specialists in perinatal mental health, mental health midwives, social workers and carers, as appropriate for patients’ individual needs. - Allocate a mental health professional to be present during the consultation for women with moderate to severe mental health conditions. | - *If we do pick up something with their mental health, you know, I would feel very obliged to make sure that they had the right kind of support. So, I would e-mail their local team to, you know, ask if they had perinatal mental health support or if not, then how we might explore going about supporting her and her family in a timely fashion. #13, Fetal cardiology specialist nurse* |

**Table 3. Individualizing care**

| **Key ideas – Recommendations** | **Quotes** |
| --- | --- |
| **Background information & setting the agenda**   - Gather information about the patient’s professional or educational background to determine the appropriate communication register and style. - Ask open-ended questions to assess the patient’s current understanding of the suspected or confirmed diagnosis. - Explore the patient’s expectations for the consultation at the outset and establish a clear agenda. | - *So, I normally start with a question “Can I please ask you to repeat in your own words what people have explained to you about the condition that the baby may or may not have” I think it's very important to understand the level of conversation that they have and their level of understanding, before I start adding info on to that. # 6, FM consultant* - *It's about understanding what you're trying to achieve. So, setting expectations for that counselling… setting the background information with the family, understanding what they're there for and what the expected outcome is… #20, Neonatal consultant* |
| **Individualized communication strategies**   - Ask the patient at regular intervals how much information they would like to receive about different aspects of their condition. - Offer the option to discuss further details later from the outset and suggest this approach if the patient appears overwhelmed. - Consider each situation holistically, considering the patient’s support systems, work patterns, and housing. - Engage the patient and their significant others in exploring available support options during challenging times. | - *People fall into 2 categories; they either going to Google everything that they can lay their hands on or they're going to stick the head in the hat and just not be able to cope with any more information. So, then it’s about finding what that person sitting in front of you needs from you. #13, Fetal cardiology specialist nurse* - *They should take into consideration the whole holistic picture. Not just about this is what's wrong with your baby here. You have to take into consideration their lifestyle at home, any support they've got, what exactly the mental health issues are. #14, FM midwife* |
| **Cultural and religious sensitivity**   - Avoid making assumptions about a patient’s preferences based on their cultural or religious background. - Present all management options to every patient clearly and impartially. - Do not revisit management options that the patient has already declined unless there is a change in the prognosis. | - *For some people even if the baby were to see the world for one second, they feel they have fulfilled their duty as a mother… #10, FM consultant* - *So, I try my best to gauge, if possible, from them on that (Opting for TOP). So if it is something they ethically don't agree with or it’s not part of their culture and they kind of lay that out for me, I don't keep mentioning it again; if that feels inappropriate for them... # 16, Fetal cardiology specialist nurse* |
| **Clinicians responding to patients' reactions**   - Be prepared for a range of emotional reactions from patients and acknowledge these as part of the grieving process. - Consider pausing consultations if patients become highly stressed or emotional, and resume discussions during a follow-up visit. - Offer second opinions if patients express disbelief, denial, or request one directly. | - *If we find that somebody's getting quite stressed and emotional, then I perhaps will hold back on the information for the first visit, and then I'll say there's a repeat scan in two weeks, let's sit down and discuss it again... #5, Paediatric surgeon* - *Think we still need to tailor make things to how they are interacting with you at the time, what's happening in front of you... #12, FM midwife* |

**Table 4.1 Teamwork**

| **Key ideas – Recommendations** | **Quotes** |
| --- | --- |
| **Roles within the team**   - Ensure the emotional and mental well-being of every patient is addressed by a member of the team. - Allocate midwives/specialist nurse to provide additional support - Consider perinatal mental health referral for patients who disclose poor mental health or display emotional distress during consultations. | - There are issues around birth and what happens after…the midwives are usually very good at dealing with those sorts of questions. #15, FM consultant - Obviously, they have a lot of bad news given to them, so it's part of our role as midwives here, to counsel them and to ensure that they are looked after well they are fully aware of what is going to happen to them (when coming for TOP), where they need to go. #14 FM midwife - And then we would bring in other people, like, you know, if it would be helpful for them to meet to meet a face-to-face psychologist, we will bring that person into our appointment or social worker or, you know anybody else that was relevant to help them support them. #13, Fetal cardiology nurse |
| **Inter/Intradisciplinary communication**   - Establish and maintain clear communication channels to ensure coordinated and efficient care across teams. This includes flagging specific patient needs upon referral (e.g., language barriers, mental health history, social circumstances) to allow for these needs to be anticipated and addressed. - Ensure patients receive consistent and comprehensive information from HCPs across different teams and specialties by conducting joint consultations. | - We also speak amongst us and say like I think I had a good shot with X. I think she understood everything. But would you mind chatting to her too? We also try to kind of cross cover each other. #6, FM consultant |

**Table 4.2 Communication training**

| **Key ideas – Recommendations** | **Quotes** |
| --- | --- |
| **Need for training**   - Establish mandatory communication training and regular refreshers for HCPs working in the fetal medicine field. - Incorporate training sessions that focus on strategies on communication with individuals with advanced needs, such as MHC. | - I feel if there was a structured training with advanced communication skills, it would be really beneficial. #10, FM consultant - So, if I know from medical history that the patient has a diagnosis of schizophrenia or severe depression with suicidal attempts, I feel very uncomfortable to touch this topic. #18, FM fellow |

**Table 4.3 Human Factors**

| **Key ideas – Recommendations** | **Quotes** |
| --- | --- |
| **Emotional impact, Coping mechanisms and compassion fatigue**   - Provide formal opportunities for sharing experiences among colleagues, such as group sessions or debriefings. - Provide emotional and mental health support within the fetal medicine units for HCPs. | - I've cried with women who've had bad news, I'm a human, right? And when you see what the woman is going through that’s really, really sad. #14, FM midwife - I have certain technique I do to leave my work stress at work and not bring home, because it's important that you disconnect... #10, FM consultant. - I think it is very easy to become just a factory of having women coming in and out of a clinic; I think we often lose the human touch when we relay messages…#12, FM midwife |

**Table 5. Health Inequalities**

| **Key ideas – Recommendations** | **Quotes** |
| --- | --- |
| **Healthcare resources**   - Allow longer consultation times to facilitate more in-depth discussions and improve healthcare professional (HCP)–patient interactions. - Provide adequate private and quiet spaces for consultations, ideally located away from busy corridors. - Explore pragmatic solutions that balance the need for high-quality patient counseling with the realities of clinic schedules (e.g., staggered appointments, dedicated consultation sessions, task shifting with other HCPs) | - So, we may counsel them, but they deliver locally. Ideally, we want to deliver in the same centre, but it's not possible all the time because of capacity issues. So, I agree they should be continuity of care, but we need to take into account the pressures for every site and be very pragmatic about it. # 20, Neonatal consultant - We've only got 1 counselling room here, so if somebody's in that already, then we have to use the scan room and we've done that. You know, it depends on the space. # 14, FM midwife - “Time is a limit in our system; if you had all the time, you might talk for an hour and a half because there's so much interaction, whereas we are driven to deliver most of it within 45 minutes” #20, neonatal consultant. |
| **Language barriers**   - Enhance access to qualified interpreters for patients with language barriers. - Avoid relying on significant others particularly when discussing ethically sensitive topics, to ensure that patients receive accurate information directly from HCPs. | - *So, I think they add a lot of content, which is a problem because sometimes you say one thing and they don't really translate verbatim. They translate, probably adding their own experience or content which doesn't help. # 6, FM consultant* - *If you've used language line, you've got somebody on the phone yes, but that's just not good at all... #15, FM consultant* |
| **Poor health literacy**   - Establish effective health education to ensure that patients understand the purpose of the interventions for which they provide consent (e.g., the aims of the ultrasound scan). - Promote and support research and public health initiatives to address health literacy gaps - Develop informative materials that explain complex conditions in lay language to enhance patient understanding. | - *So, I think often you need to simplify it, and I think even people who are extremely bright without mental health conditions cannot process information we give them in medical consultations, despite our best endeavours… # 4, Neonatal consultant* - *And I suppose the difficulty there is if she explains it back in a very, very simple language… Has she understood it? In reality, it can be quite difficult… # 2, FM fellow* |
| **Challenges around mental health support access**   - Increase the capacity of perinatal mental health services to effectively meet the needs of patients. - Enhance awareness of mental health conditions to reduce the stigma associated with these issues. | - *I think there is a lot of stigma around mental health and women usually are not very keen to talk about those issues… #18, FM fellow* - *For people who have minor Mental health problems, It's probably more difficult 'cause they don’t have an established support network. I cannot pretend to be a counselor or a mental health specialist... # 1, FM consultant* |
